# Inter-rater Agreement of Richmond Agitation Sedation Scale Assessments in Adult Patients Receiving Mechanical Ventilation in the ICU: A Cross-Sectional Study

**DOI:** 10.1097/CCE.0000000000001302

**Published:** 2025-08-28

**Authors:** Mikita Fuchita, Jack Pattee, David Le, Tien To, Carlos Mucharraz, Sara Knippa, Alexis Keyworth, Caitlin Blaine, Heidi Lindroth

**Affiliations:** 1 Department of Anesthesiology, University of Colorado Anschutz Medical Campus, Aurora, CO.; 2 Department of Biostatistics and Informatics, Center for Innovative Design & Analysis, Colorado School of Public Health, Aurora, CO.; 3 University of Colorado School of Medicine, Aurora, CO.; 4 UCHealth University of Colorado Hospital, Aurora, CO.; 5 Department of Surgery, Division of Cardiothoracic Surgery, University of Colorado Anschutz Medical Campus, Aurora, CO.; 6 Division of Nursing Research, Department of Nursing, Mayo Clinic, Rochester, MN.; 7 Center for Aging Research, Regenstrief Institute, Center for Health Innovation and Implementation Science, School of Medicine, Indiana University, Indianapolis, IN.

**Keywords:** health services research, mechanical ventilation, nursing care, psychomotor agitation, quality improvement, sedation

## Abstract

**IMPORTANCE:**

Accurate and reliable sedation assessment is crucial to improving patient outcomes in the ICU.

**OBJECTIVE:**

To evaluate the inter-rater agreement and reliability of Richmond Agitation Sedation Scale (RASS) assessments between bedside nurses and trained investigators in patients receiving mechanical ventilation in the ICU.

**DESIGN, SETTING, AND PARTICIPANTS:**

An assessor triad, comprising an ICU nurse providing direct patient care and two trained investigators, simultaneously performed RASS assessments during 79 encounters with 62 unique patients receiving mechanical ventilation at two ICUs at a tertiary care academic hospital in Colorado. A total of 58 nurses participated in the study.

**MAIN OUTCOMES AND MEASURES:**

The inter-rater reliability of RASS assessments was evaluated with the intraclass correlation coefficient (ICC) and weighted kappa (κ), and inter-rater agreement was evaluated with percentage agreement and Bland-Altman analysis.

**RESULTS:**

Acute respiratory failure (55%) and altered mental status (21%) were the most common reasons for mechanical ventilation. Most patients were receiving one (interquartile range, 0.5–2) continuous sedative during the assessment. The inter-rater reliability of RASS assessments between the nurses and investigators (ICC, 0.728–0.779; weighted κ, 0.62–0.63) was lower than between the two investigators (ICC, 0.891; weighted κ, 0.80). The assessor triad agreed on the same RASS values in only 35% of observations. The average differences in RASS were greater between the investigators and nurses, ranging from –0.658 to –0.544, compared with 0.114 between the two investigators. Compared with the mean of the two investigators, RASS values recorded by nurses were more likely to be higher (52% of observations), indicating a lighter sedation level. In 16% of observations, at least one assessor commented on uncertainty or ambiguity with the RASS.

**CONCLUSIONS AND RELEVANCE:**

The inter-rater reliability of RASS assessments was high. However, we observed variations in the degree of agreement by assessor category. Further studies are necessary to explore how factors such as assessor characteristics, ICU environment, and patient conditions influence the inter-rater agreement of the RASS in contemporary ICU practices.

**KEY POINTS**

**Question**: What is the inter-rater agreement and reliability of Richmond Agitation Sedation Scale (RASS) assessments between bedside nurses and trained investigators in adult patients receiving mechanical ventilation in the ICU?

**Findings**: This cross-sectional study found high inter-rater reliability of RASS assessments between nurses and trained investigators. However, the assessor triad, comprising a nurse and two trained investigators, agreed on the same RASS values in only 35% of observations, and we observed variations in the degree of agreement by the assessor category.

**Meaning**: Although the inter-rater reliability of RASS assessments between nurses and the trained investigators was high, further studies are necessary to explore how factors such as assessor characteristics, ICU environment, and patient conditions influence the inter-rater agreement of the RASS.

Over 2 decades have passed since the introduction of the Richmond Agitation Sedation Scale (RASS) (1, 2). The RASS has become the standard bedside tool for evaluating the depth of sedation among adult patients in the ICU (3). The availability of a validated, implementable, and scalable sedation assessment tool has facilitated research investigating the impact of sedation practices on patient outcomes. There is now clear evidence that deep sedation, defined as RASS less than or equal to –2 in recent literature (3–6), is associated with increased risks for delirium, post-traumatic stress disorder, prolonged immobilization, delayed extubation, and death (5, 7–9). Sedation optimization is a key evidence-based intervention to improve patient outcomes in the ICU (3, 10–12).

Despite the initial validation studies of the RASS in the early 2000s, a recent study raised concerns that RASS assessments by bedside nurses may be susceptible to measurement biases; the median RASS recorded by nurses and independent investigators was –2 and –4.5, respectively (*p* = 0.003) (13). However, this study had a small sample size (*N* = 21) and used 38 paired observations of RASS assessments performed up to 4 hours apart, which may have exaggerated the observed discrepancies. Given the increasing importance of accurate and reliable RASS assessments, we evaluated the inter-rater agreement and reliability of RASS assessments between ICU nurses providing direct patient care and trained investigators in current ICU practices.

## MATERIALS AND METHODS

### Study Design and Setting

This was a single-center cross-sectional study of RASS assessments performed by 58 bedside ICU nurses and three trained investigators at Medical and Cardiothoracic ICUs within a tertiary care academic hospital in Colorado. Both are 24-bed ICUs that manage patients with diverse medical conditions and high acuity levels. Bedside nurses who work in these ICUs receive formal training in RASS assessment as part of a 1-hour session on the ABCDEF bundle led by a clinical nurse specialist. All nurses new to ICU level of care attend this session during their orientation, and the return demonstration is completed during their preceptorship in the ICU. As part of routine care, they are expected to document a RASS value every two to four hours for all patients in the ICU. In addition, these ICUs follow the system-wide ICU Liberation ABCDEF bundle protocols (10) and perform the Spontaneous Awakening Trial and Spontaneous Breathing Trial during the early morning hours, typically around 8 am. most patients have a target RASS of –1 to +1 unless they have specific medical indications for deep sedation, as determined by the providers.

This study was part of a quality improvement effort to understand local ICU sedation practices, and the Colorado Multiple Institutional Review Board determined it to be exempt from review (number 22-0223). Since patients without an oral endotracheal tube do not routinely receive sedation in these ICUs, this project examined only those receiving mechanical ventilation with an oral endotracheal tube. The reporting of this study adhered to the Strengthening the Reporting of Observational Studies in Epidemiology statement (**eTable 1**, https://links.lww.com/CCX/B542) (14).

### Data Collection

In preparation, three investigators (two medical students and a resident physician) received a 90-minute training on the RASS from a board-certified intensivist, including a lecture and bedside training in the Cardiothoracic ICU. To qualify as trained investigators, they were required to pass a 10-question video RASS assessment quiz with greater than or equal to 90% accuracy and agree with RASS assessments on three consecutive patients receiving mechanical ventilation. The standard definition and procedure of the RASS described by Sessler et al (1) were used for the training. Two of the three trained investigators were paired to perform the RASS with nurses, as detailed below.

Between May 2024 and August 2024, we used convenience sampling to identify patients receiving mechanical ventilation with an oral endotracheal tube and approached nurses caring for these patients for study participation. The study team explained to them that the objective was to understand the reliability of the RASS and not to evaluate the performance of individual nurses. To further minimize biases, the study team reassured the nurses that their responses were anonymous and confidential. For this reason, we did not collect their demographics or professional background data. Study participation was voluntary, and nurses were encouraged to decline for any reason. Repeat study participation was tracked by obtaining the last four digits of their employee number. Aside from refusal by nurses or family members, no patients were excluded based on their medical conditions.

For the RASS assessments, two trained investigators and the patient’s bedside nurse comprised an assessor triad. They entered the room together to perform a RASS assessment. The assessment began by silently observing the patient, followed by verbal stimulation, calling the patient’s name and asking them to open their eyes and look at the speaker. If the patient remained unresponsive, one of the assessors provided physical stimulation, shaking the shoulder and progressing to a noxious stimulus, as needed. The patient was stimulated (verbally and/or physically) only once, which informed the three independent RASS assessments (i.e., interpretation of the response was independent). A professional interpreter was used if the patient’s primary spoken language was non-English.

Each encounter concluded after the three assessors independently recorded their RASS assessments on a secure online server. Nurses were instructed to report the RASS as they would in usual care, and their data entry form mirrored the format of their clinical documentation (**eFig. 1**, https://links.lww.com/CCX/B542). Whenever the assessors felt uncertain or ambiguous about a patient’s RASS, they could submit comments to describe it. The investigators also recorded the continuous sedatives at the time of the assessment, which were not paused or adjusted for these RASS assessments.

Patient demographics, comorbidities, reasons for mechanical ventilation, length of stay, and 90-day mortality were extracted through a retrospective chart review. Investigators who extracted the electronic health record data were blinded to the results of the RASS assessments.

### Statistical Analysis

Patient characteristics, sedative data, and recorded RASS were summarized using descriptive statistics. The RASS is an ordinal scale, meaning that while the values indicate rank order, the intervals between them are not necessarily equal or quantitatively meaningful. Still, it was originally introduced as a scale that may be treated as a continuous variable (1). Therefore, to enhance the robustness of our statistical analysis, we used both the intraclass correlation coefficient (ICC) and weighted kappa (𝜅) (Cicchetti-Allison equal spacing weights) to evaluate inter-rater reliability and Bland-Altman analysis to evaluate agreement and measurement biases, reported as mean difference between two comparators and limits of agreement (the 2.5th percentile and 97.5th percentile of the distribution of differences). We used two-tailed testing with an alpha threshold of 0.05 for statistical significance. The study was designed to be exploratory, and a hypothesis-driven power calculation was not performed; to characterize the inter-rater reliabilities and the Bland-Altman relationships, we determined a priori that a convenience sample of 100 observations would suffice. The study concluded after 79 observations due to investigator’s availability. There were no missing values. We used the statistical software R, Version 4.4.2 (R Foundation for Statistical Computing, Vienna, Austria) for analysis and data visualization.

## RESULTS

Of 96 eligible patient encounters, 79 had RASS assessments completed by the assessor triad (**eFig. 2**, https://links.lww.com/CCX/B542). As shown in **Table 1**, the sample included 62 unique patients with a mean age of 56 (sd, 16.2) years, 44% female, and 66% White. Acute respiratory failure (55%) and altered mental status (21%) were the most common reasons for mechanical ventilation. The median (interquartile range [IQR]) Acute Physiology and Chronic Health Evaluation II Score was 24 (IQR, 19.2–28). The RASS assessments occurred on a median of 3.5 (IQR, 1–10) days after initiation of mechanical ventilation, mostly during the hours between 10 am and 4 pm (77%, 61/79; eTable 2, https://links.lww.com/CCX/B542). Most had a target RASS of –1 to +1 (72%, 57/79) and were receiving one (IQR, 0.5–2) continuous sedative infusion at the time of the assessment, with propofol (48%) and fentanyl (44%) being the most frequently used sedatives (**eTable 3**, https://links.lww.com/CCX/B542). Fifty-eight nurses participated in the study, most only once (71%, 41/58), and some twice (24%, 14/58), three times (3%, 2/58), and four times (2%, 1/58).

**TABLE 1.** Patient Characteristics

Variables	Medical ICU (*n* = 38)	Cardiothoracic ICU (*n* = 24)	Overall (*n* = 62)
Age (yr)	54 (17.4)	60 (13.5)	56 (16.2)
Sex (female)	19 (50.0%)	8 (33.3%)	27 (43.5%)
Race			
White	25 (65.8%)	16 (66.7%)	41 (66.1%)
Black	8 (21.1%)	3 (12.5%)	11 (17.7%)
American Indian or Alaska Native	0	2 (8.3%)	2 (3.2%)
Other	5 (13.2%)	3 (12.5%)	8 (12.9%)
Hispanic	9 (23.7%)	3 (12.5%)	12 (19.4%)
Primary spoken language			
English	34 (89.5%)	21 (87.5%)	55 (88.7%)
Spanish	3 (7.9%)	2 (8.3%)	5 (8.1%)
Other	1 (2.6%)	1 (4.2%)	2 (3.2%)
Body mass index (kg/m^2^)	27.6 (7.1)	30.4 (9.9)	28.7 (8.3)
Charlson comorbidity index	5.0 (3.0–8.0)	5.0 (3.0–6.0)	5.0 (3.0–6.8)
Anxiety or depression	14 (36.8%)	10 (41.7%)	24 (38.7%)
Post-traumatic stress disorder	4 (10.5%)	2 (8.3%)	6 (9.7%)
Alcohol abuse disorder	6 (15.8%)	1 (4.2%)	7 (11.3%)
Cognitive impairment	6 (15.8%)	1 (4.2%)	7 (11.3%)
Hearing or visual impairment	4 (10.5%)	3 (12.5%)	7 (11.3%)
Cerebral vascular accident	10 (26.3%)	7 (29.2%)	17 (27.4%)
Chronic obstructive pulmonary disease	6 (15.8%)	6 (25.0%)	12 (19.4%)
Chronic liver disease	10 (26.3%)	3 (12.5%)	13 (21.0%)
Chronic kidney disease	10 (26.3%)	6 (25.0%)	16 (25.8%)
Reason for mechanical ventilation			
Acute respiratory failure	25 (65.8%)	9 (37.5%)	34 (54.8%)
Altered mental status	9 (23.7%)	4 (16.7%)	13 (21.0%)
Emergency procedure	2 (5.3%)	4 (16.7%)	6 (9.7%)
Postoperative respiratory failure	0	6 (25.0%)	6 (9.7%)
Hemodynamic instability	2 (5.3%)	1 (4.2%)	3 (4.8%)
Acute Physiology and Chronic Health Evaluation II Score	23.5 (18.2–28)	24 (22–29.2)	24 (19.2–28)
ICU length of stay (d)	12 (6–19.5)	14 (5.5–21.5)	13 (6–20.8)
Hospital length of stay (d)	17.5 (12–38)	26.5 (12.8–39.5)	20 (12–38.8)
90-d mortality	21 (55.3%)	8 (33.3%)	29 (46.8%)

Values are described as count (%), mean (sd), or median (interquartile range) as appropriate based on data type and distribution.

**Figure 1.**
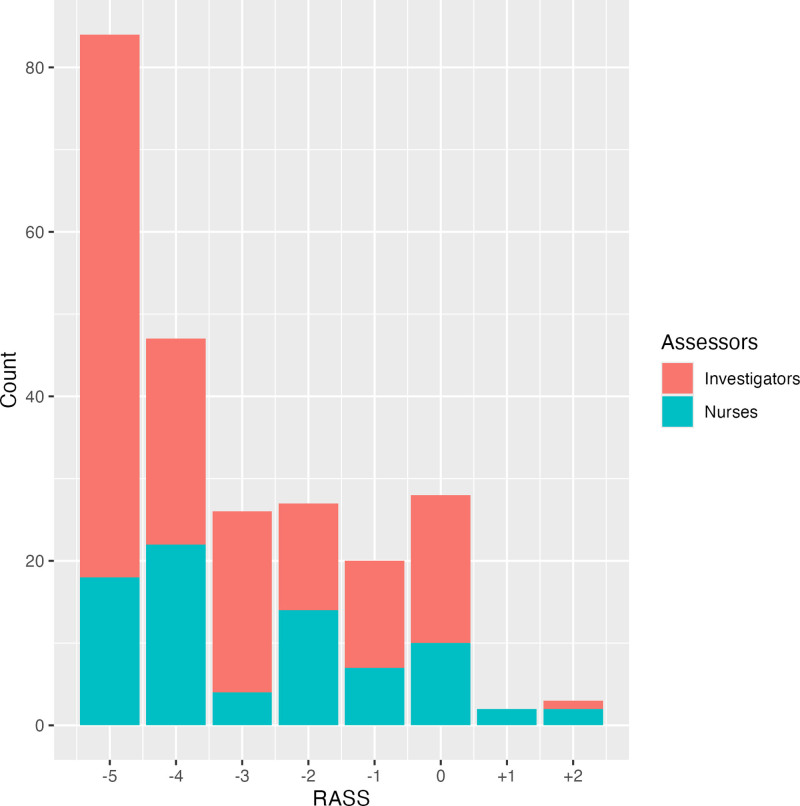
Distribution of Recorded Richmond Agitation Sedation Scale (RASS). A stacked histogram of all recorded RASS (*n* = 237) by the three assessors. *Red* indicates those recorded by the two trained investigators combined. *Blue* indicates those recorded by the nurses. The distribution of RASS was skewed with a high frequency of recorded RASS in the negative values.

**Figure 1** illustrates the distribution of all recorded RASS. Among the recorded RASS values, –5 was the most common (35%, 84/237). There was no RASS +3 or +4. The two investigators agreed on the same RASS values in 70% (55/79) of observations. The assessor triad agreed on the same RASS values in 35% (28/79) of observations. Compared with the mean RASS values recorded by the two investigators, RASS values recorded by nurses were higher (indicating a lighter sedation level) in 52% (41/79) and lower (indicating a deeper sedation level) in 13% (10/79) of observations.

**Figure 2.**
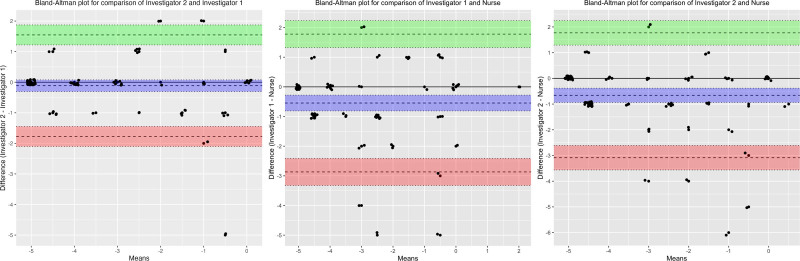
Bland-Altman Plot Comparing Trained Investigators and Nurses. Bland-Altman bias estimation using the Richmond Agitation Sedation Scale (RASS) assessments from the overall sample (79 observations): comparison of investigators 1 and 2 (**left figure**), comparison of investigator 1 and nurse (**middle figure**), and comparison of investigator 2 and nurse (**right figure**). The *purple bands* represent the bias estimate and their CI. The *green bands* represent the upper limits of agreement and their CI. The *red bands* represent the lower limits of agreements and their CI. *Bands* closer to 0 indicate a higher level of agreement in RASS assessments between assessors.

As shown in **Table 2**, the overall inter-rater reliability between the nurses and investigators (ICC, 0.728–0.779; weighted 𝜅, 0.62–0.63) was lower than between the two investigators (ICC, 0.891; weighted 𝜅, 0.80). When stratified by ICU types, the inter-rater reliability between the nurses and investigators in the Cardiothoracic ICU (ICC, 0.729–0.859; weighted 𝜅, 0.65–0.70) was higher than the Medical ICU (ICC, 0.653–0.670; weighted 𝜅, 0.51–0.52). As shown in **Figure 2** and **Table 3**, the overall mean differences in RASS values were close to zero (within one sedation level), indicating no major systematic bias. When compared by the assessor category, the mean difference between the investigators and nurses, which ranged from –0.658 (–0.936 to –0.381; *p* < 0.001) to –0.544 (–0.810 to –0.279; *p* < 0.001), was greater than 0.114 (–0.076 to 0.304, *p* = 0.24) between the two investigators. The Bland-Altman plot showed funnel-like distributions, whereas greater agreement was observed in the lowest mean RASS values (around –5 to –4), with increasing variability in higher mean RASS values (around –3 to 0) (Fig. 2).

**TABLE 2.** Inter-rater Reliability of Richmond Agitation Sedation Scale Assessments

Patient Population	Intraclass Correlation Coefficient (95% CI)	Weighted κ (95% CI)
Overall sample (*n* = 79)		
Investigator 1 vs. 2	0.891 (0.836–0.929)	0.80 (0.72–0.88)
Investigator 1 vs. nurses	0.779 (0.675–0.853)	0.63 (0.53–0.74)
Investigator 2 vs. nurses	0.728 (0.606–0.817)	0.62 (0.50–0.73)
Mean investigators vs. nurses	0.773 (0.667–0.849)	NA
Medical ICU (*n* = 50)		
Investigator 1 vs. 2	0.897 (0.826–0.940)	0.76 (0.67–0.85)
Investigator 1 vs. nurses	0.653 (0.461–0.786)	0.51 (0.38–0.64)
Investigator 2 vs. nurses	0.662 (0.474–0.793)	0.52 (0.37–0.67)
Mean investigators vs. nurses	0.670 (0.484–0.798)	NA
Cardiothoracic ICU (*n* = 29)		
Investigator 1 vs. 2	0.855 (0.717–0.929)	0.79 (0.64–0.95)
Investigator 1 vs. nurses	0.859 (0.724–0.931)	0.70 (0.56–0.84)
Investigator 2 vs. nurses	0.729 (0.503–0.862)	0.65 (0.47–0.84)
Mean investigators vs. nurses	0.831 (0.673–0.916)	NA

NA = not applicable.

**TABLE 3.** Bland-Altman Analysis of Richmond Agitation Sedation Scale Assessments

Patient Population	Bias	95% CI	*p*	Lower LoA	Upper LoA
Overall sample (*n* = 79)					
Investigator 1 vs. 2	0.114	–0.076 to 0.304	0.235	–1.546	1.774
Investigator 1 vs. nurses	–0.544	–0.810 to –0.279	< 0.001	–2.868	1.779
Investigator 2 vs. nurses	–0.658	–0.936 to –0.381	< 0.001	–3.087	1.771
Mean investigators vs. nurses	–0.601	–0.856 to –0.347	< 0.001	–2.828	1.626
Medical ICU (*n* = 50)					
Investigator 1 vs. 2	0.060	–0.125 to 0.245	0.518	–1.218	1.338
Investigator 1 vs. nurses	–0.580	–0.948 to –0.212	0.002	–3.118	1.958
Investigator 2 vs. nurses	–0.640	–0.993 to –0.287	< 0.001	–3.073	1.793
Mean investigators vs. nurses	–0.610	–0.958 to –0.262	0.001	–3.013	1.793
Cardiothoracic ICU (*n* = 29)					
Investigator 1 vs. 2	0.207	–0.217 to 0.631	0.325	–1.977	2.391
Investigator 1 vs. nurses	–0.483	–0.858 to –0.108	0.014	–2.416	1.451
Investigator 2 vs. nurses	–0.690	–1.168 to –0.212	0.006	–3.152	1.773
Mean investigators vs. nurses	–0.586	–0.960 to –0.212	0.003	–2.512	1.340

LoA = limits of agreement, the 2.5th percentile and 97.5th percentile of the distribution of differences.

Bias is calculated by averaging the differences between the two comparators. For example, a bias of –0.544 for “investigator 1 vs. nurses” in the overall sample is calculated as the average of (Richmond Agitation Sedation Scale [RASS] recorded by investigator 1) – (RASS recorded by nurses). *p* < 0.05 indicates that there was a statistically significant difference between the recorded RASS between the assessors (i.e., the null hypothesis is that there is no significant difference in recorded RASS between the assessors).

Thirteen of 79 (16%) observations had comments by at least one of the three assessors describing uncertain or ambiguous RASS assessments (**eTable 4**, https://links.lww.com/CCX/B542). Four were related to the interpretation of eye-opening (e.g., following commands appropriately but unable to open eyes due to severe edema), three were related to the type of noxious stimuli used for RASS assessments (e.g., whether to include tracheal suction or not), and two were related to uncertainties determining the RASS in patients with involuntary movements. The remaining four comments pertained to the temporal swings in patients’ states, where assessors had to assign a single RASS value despite the patients’ fluctuating condition (e.g., swinging between RASS +2 and –2 depending on the stimulus level). In a post hoc analysis excluding these 13 “difficult” cases (*n* = 66), inter-rater reliability was higher than in the overall sample, with improved reliability between the nurses and investigators (ICC, 0.826–0.842; weighted 𝜅, 0.68–0.69) and between the two investigators (ICC, 0.939; weighted 𝜅, 0.83).

## DISCUSSION

In this single-center study of critically ill patients receiving mechanical ventilation in Medical and Cardiothoracic ICUs, we found high inter-rater reliability of RASS assessments between bedside nurses and trained investigators, though agreement was not perfect. In the overall sample, the ICCs ranged from 0.728 to 0.891 and weighted 𝜅 values from 0.62 to 0.80, with greater reliability between the two investigators than between nurses and investigators. Furthermore, only 35% of RASS assessments were concordant across the full assessor triad, and nurses were more likely than investigators to assign higher RASS values, indicating lighter sedation, with an average difference of 0.6. These findings may represent potential biases and limitations of the RASS in current ICU practices, which warrant further discussion.

The reliability and validity of the RASS were first tested by Sessler et al (1) and Ely et al (2). In the first study (1), the inter-rater reliability was excellent, with ICC of 0.956 and weighted 𝜅 of 0.73 in study phase 1, comprising of simultaneous RASS assessments among five investigators (two physicians, two nurses, and one pharmacist), and ICC of 0.964 and weighted 𝜅 of 0.80 in study phase 2 between a nurse educator and 27 bedside nurses. Notably, all five investigators in study phase 1 selected the same RASS values in 60.4% of 192 observations despite including ICU populations more diverse than in our present study. In the second study (2), the weighted 𝜅 was 0.91 between two nurse investigators who simultaneously observed 290 patients in adult Medical and Coronary ICUs, indicating excellent inter-rater reliability. Interestingly, the inter-rater reliability was lower when they compared the RASS assessments by the nurses with an intensivist and a neuropsychiatric expert, with the weighted 𝜅 ranging from 0.79 to 0.88. However, these values were not derived from RASS assessments performed simultaneously, but occurring within 4 hours apart. In both studies, the inter-rater reliability was equally high in patients with and without mechanical ventilation (1, 2).

Our study also demonstrated high inter-rater reliability of the RASS, but the degree of agreement was not as robust. Rather, our study findings raised similar concerns about the RASS described in recent studies. In a small study of 21 patients in the Medical ICU with 38 observations, the concordance between RASS assessments by nurses and independent investigators was poor (𝜅 = 0.21), with a median RASS of –2 vs. –4.5, respectively (*p* = 0.003) (13). Importantly, this study used RASS values documented as part of routine nursing care in the analysis instead of simultaneous assessments. In another study reported as a conference abstract, the weighted 𝜅 between RASS values documented by nurses and those performed by a reference rater was 0.281 (15). The majority of the RASS assessments by the reference rater were between –3 and –5, whereas those charted by the nurses often reflected lighter sedation levels with RASS between –2 and 0. In both studies, RASS values recorded by bedside nurses were higher than their references (13, 15), which was also the case in another study examining the Ramsay Sedation Scale, a similar sedation scale (16).

Compared to these previous studies, we followed a more rigorous study method using simultaneous RASS assessments (similar to the initial validation studies) and included more patient samples. The degree of discordance was not as prominent in our study, but we also observed that the RASS recorded by nurses was frequently higher than the trained investigators. To explain this, we speculate on the influences of several cognitive biases.

First is recency bias. In our study, investigators received rather rigorous training using the formal definitions and procedure of the RASS immediately before the study, whereas the bedside nurses did not receive such additional training. As this study was part of a quality improvement effort to understand the local ICU practices, we intentionally omitted additional guidance for nurses and made efforts so that data obtained from them reflected their usual practices. It is worth noting that while the investigators received recent training, they did not have the same level of bedside experience or familiarity with the RASS as the nurses—it is debatable who the true expert was. The second is biases due to priming. While the investigators evaluated each patient on single occasions, nurses were present with their patients throughout their entire shift. Although the nurses and investigators performed RASS assessments simultaneously, RASS recorded by nurses could have been influenced by their prior patient encounters without their conscious awareness. Lastly, commitment bias might have been present among nurses because they were also responsible for titrating sedatives to a goal sedation level set by the providers, which was RASS –1 to +1 in 72% of our cases. This may explain the direction of bias observed in our study, consistent with all previous studies (13, 15, 16).

Based on the comments provided by our assessors in 16% of observations, we also identified potential limitations of the RASS. First, over time, users may adopt definitions and procedures of the RASS that are different from those originally described, such as replacing sternal rub with tracheal suctioning. Second, due to the reliance on eye-opening and gaze, the RASS may falsely categorize patients in the deeper sedation categories when they are unable to open their eyes. Conversely, a comatose patient would receive RASS –3 (instead of –5) if their eyes are open, albeit involuntarily. And third, patients’ level of arousal is known to fluctuate over time, sometimes in a matter of seconds to minutes (2), “swinging from RASS –2 to +2,” as noted by one nurse. Regardless, nurses (or investigators in research studies) are required to assign a single RASS value per patient assessment, introducing room for subjectivity. To overcome this challenge, previous research has explored electroencephalography-based measures like the bispectral index to continuously and objectively quantify the depth of sedation (3, 17). However, these tools come with their own limitations and are not widely used in standard practice.

Another noteworthy finding in our study is the differences in inter-rater reliability between nurses and investigators by ICU type. Namely, inter-rater reliability between nurses and investigators was higher in the Cardiothoracic ICU compared with the Medical ICU. Although available data cannot directly explain these differences, patient population and unit culture (such as shared assumptions, values, and goals) may shape how the RASS is performed in each unit (18).

Finally, only 5 of 237 (2%) of all recorded RASS were in the positive range. This finding is similar to the initial validation studies by Sessler et al and Ely et al (1, 2) and is likely attributable to the overuse of sedation in the ICU. We also believe this reflects the safety concerns shared among nurses that patients manifesting restlessness and agitation may fall or accidentally remove vascular accesses and surgical drains, prompting immediate interventions to calm them. In our study, 10 nurses declined study participation due to being busy with clinical tasks (eFig. 2, https://links.lww.com/CCX/B542). Some of these may have been due to attending to patients who were restless or agitated, contributing to a selection bias.

The major strengths of this study are 1) the use of two independent investigators to determine the degree of bias among trained assessors, 2) rigorous training of investigators using the original RASS definitions and procedure, 3) simultaneous RASS assessments by the investigators and nurses, and 4) the use of robust statistical analytics to quantify the scale reliability and measurement biases.

There are several important study limitations. First, this was a single-center study. Although the inclusion of two distinct ICUs increased patient diversity, our findings may not be generalizable to other settings, such as non-academic ICUs and those serving different patient populations. Second, the sample size was relatively small, and the use of convenience sampling may have introduced unintended selection biases. Third, we did not collect detailed information about the participating nurses, such as age, sex, education and training background, years of ICU experience, and employment status (e.g., hospital employee vs. travel nurse). Differences in these characteristics and how the investigators and nurses were trained on the RASS may have contributed to the variations in inter-rater reliability and agreement observed in this study. Fourth, while the previous validation study treated the RASS as a continuous variable (1), it is fundamentally an ordinal scale. For this reason, we evaluated inter-rater reliability using both the ICC and weighted 𝜅, and we advise caution in interpreting the results of the ICC and Bland-Altman analysis given the scale’s ordinal nature. Lastly, to improve internal validity, we collected RASS data through simultaneous RASS assessments rather than using those documented in routine clinical care. As such, our findings may not fully reflect how the RASS is recorded in everyday clinical practice. Despite these limitations, our study contributes important new data on the inter-rater agreement and reliability of the RASS, highlighting the need for further research to explore how factors such as assessor characteristics, ICU environment, and patient conditions may influence RASS assessments in real-world settings.

## CONCLUSIONS

The inter-rater reliability of bedside RASS assessments between nurses and the trained investigators was high. However, we observed variations in the degree of agreement by the assessor category. Although this study supports the continued use of the RASS as a reliable sedation assessment tool in adult patients receiving mechanical ventilation in the ICU, further studies are necessary to explore how different factors may influence its inter-rater agreement.

## ACKNOWLEDGMENTS

We thank Maureen Hall, BSN, RN, Mark Yoder, BSN, RN, CCRN, Erica Pratt, BSN, RN, CCRN, and bedside nurses at the study site ICUs for graciously cooperating in the data collection despite their busy clinical workload. We are also grateful to the University of Colorado Department of Anesthesiology and the Institute for Healthcare Quality, Safety, and Efficiency for supporting this project.

## Supplementary Material
