# Supplementary material for: Inter-rater Agreement of Richmond Agitation Sedation Scale Assessments in Adult Patients Receiving Mechanical Ventilation in the ICU: A Cross-Sectional Study
Source: Crit Care Explor. 2025 Aug 28;7(9):e1302. doi: 10.1097/CCE.0000000000001302 (PMC12398364; doi:10.1097/CCE.0000000000001302)
Supplement: Supplementary file 1 [file cc9-7-e1302-s001.pdf]

Supplemental Digital Contents

Title: Inter-rater Agreement of Richmond Agitation Sedation Scale Assessments in Adult Patients Receiving Mechanical Ventilation in the Intensive Care Unit: A Cross-Sectional Study

Authors: Mikita Fuchita, MD[1]; Jack Pattee, PhD[2]; David Le, BS[3]; Tien To, BS, MS[3]; Carlos Mucharraz, MD[1]; Sara Knippa, MS, RN, CCRN, PCCN, ACCNS-AG[4]; Alexis Keyworth, MSHS, PA-C[5]; Caitlin Blaine, MSHS, PA-C[5]; Heidi Lindroth, PhD, RN, FAAN[6, 7].

Affiliations:
1. Department of Anesthesiology, University of Colorado Anschutz Medical Campus, Aurora, CO.
2. Department of Biostatistics & Informatics, Center for Innovative Design & Analysis, Colorado School of Public Health, Aurora, CO.
3. University of Colorado School of Medicine, Aurora, CO.
4. UCHealth University of Colorado Hospital, Aurora, CO.
5. Department of Surgery, Division of Cardiothoracic Surgery, University of Colorado Anschutz Medical Campus, Aurora, CO.
6. Division of Nursing Research, Department of Nursing, Mayo Clinic, Rochester, MN, USA
7. Center for Aging Research, Regenstrief Institute, Center for Health Innovation and Implementation Science, School of Medicine, Indiana University, Indianapolis, IN, USA

Corresponding Author:
Mikita Fuchita, MD
CU Anschutz Leprino Building
12401 East 17th Avenue, 7th Floor
Aurora, CO 80045
Email: mikita.fuchita@cuanschutz.edu
This work was conducted at the University of Colorado Anschutz Medical Campus, Aurora, CO.

TABLE OF CONTENTS

eFigure 1. Data Entry Forms for Investigators (A) and Nurses (B)………………… Pages 3-4
eFigure 2. Enrollment Flow Diagram…………………………………………….…... Page 5
eTable 1. STROBE Checklist………………………………………………………... Pages 6-8
eTable 2. Richmond Agitation Sedation Scale Assessment Time of Day ……..….. Page 9
eTable 3. Sedative Infusions at the Time of Richmond Agitation Sedation Scale Assessments …………………………………………………………………….. Page 10
eTable 4. Comments on Richmond Agitation Sedation Scale Provided by Independent Assessors ...…………………………………………………..…….. Page 11

eFigure 1. Data Entry Forms for Investigators (A) and Nurses (B)

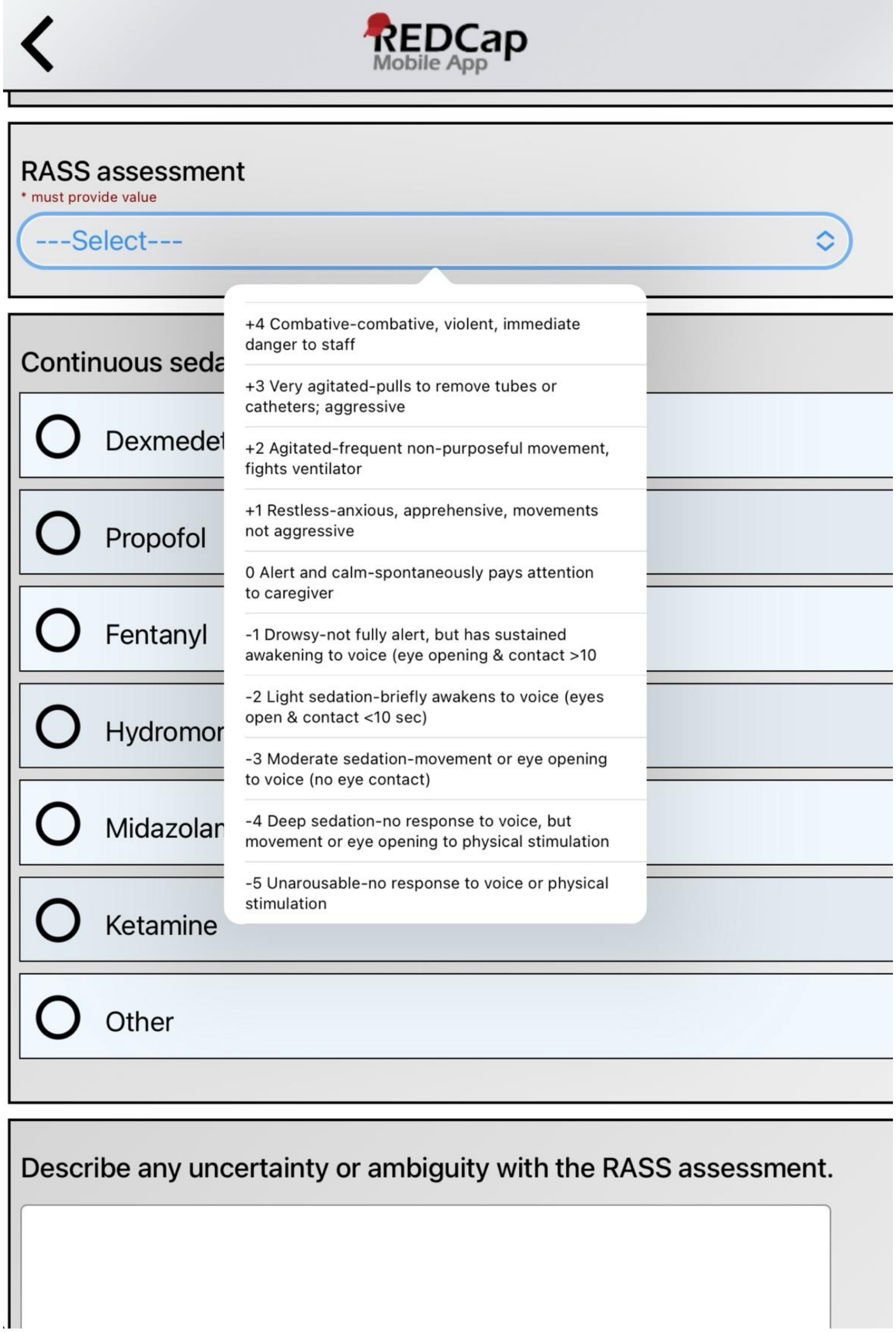
REDCap
Mobile App

RASS assessment
* must provide value
---Select---

+4 Combative-combative, violent, immediate danger to staff
+3 Very agitated-pulls to remove tubes or catheters; aggressive
+2 Agitated-frequent non-purposeful movement, fights ventilator
+1 Restless-anxious, apprehensive, movements not aggressive
0 Alert and calm-spontaneously pays attention to caregiver
-1 Drowsy-not fully alert, but has sustained awakening to voice (eye opening & contact >10
-2 Light sedation-briefly awakens to voice (eyes open & contact <10 sec)
-3 Moderate sedation-movement or eye opening to voice (no eye contact)
-4 Deep sedation-no response to voice, but movement or eye opening to physical stimulation
-5 Unarousable-no response to voice or physical stimulation

Continuous seda
Dexmedet
Propofol
Fentanyl
Hydromor
Midazolar
Ketamine
Other

Describe any uncertainty or ambiguity with the RASS assessment.

eFigure 1A: REDCap Mobile App data entry form used for the investigators, which included detailed definitions of the Richmond Agitation Sedation Scale as described by Sessler et al.

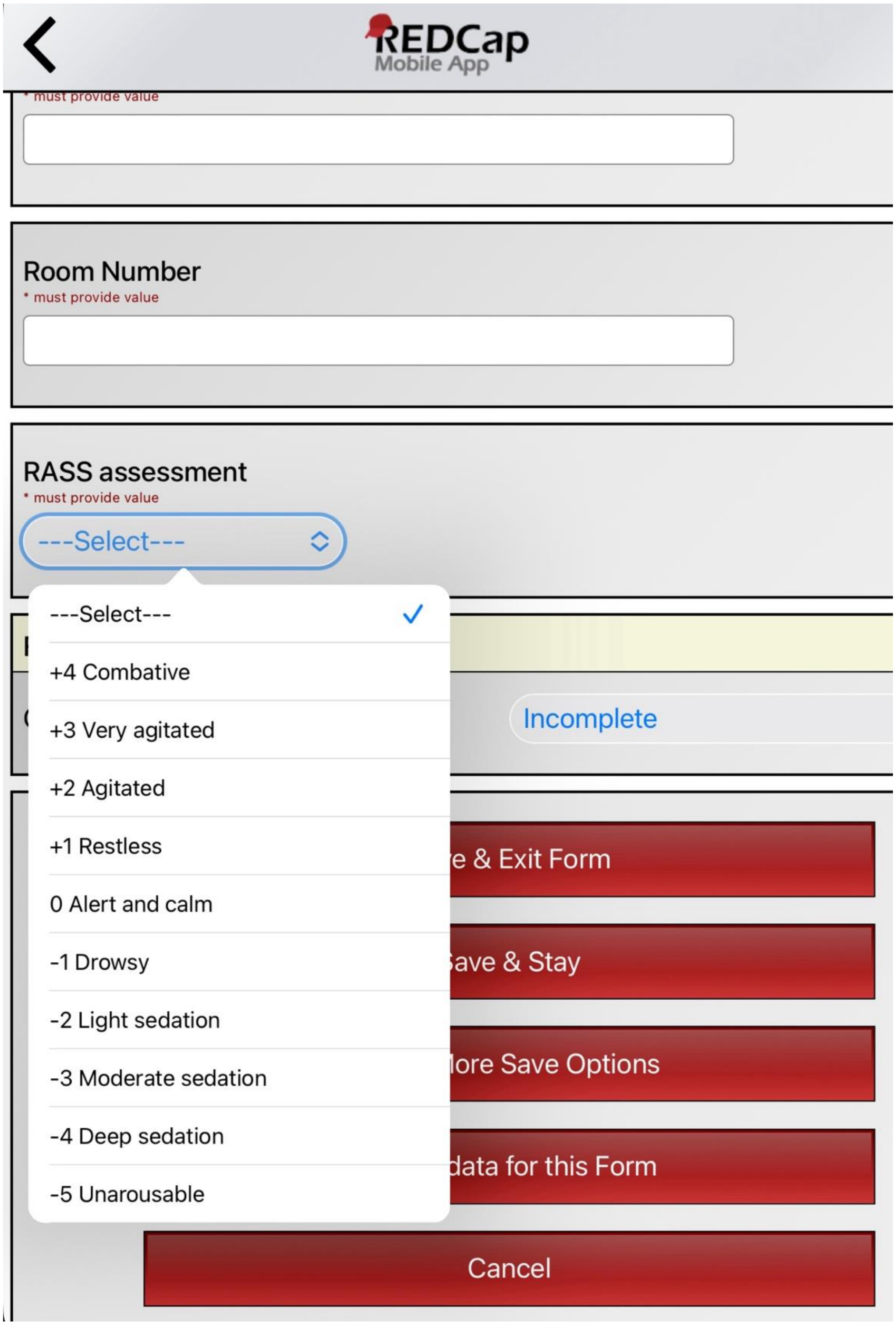

eFigure 1B: REDCap Mobile App data entry form used for the nurses, which included limited descriptions of each Richmond Agitation Sedation Scale, mirroring the electronic health record charting system used by the bedside nurses.

eFigure 2. Enrollment Flow Diagram

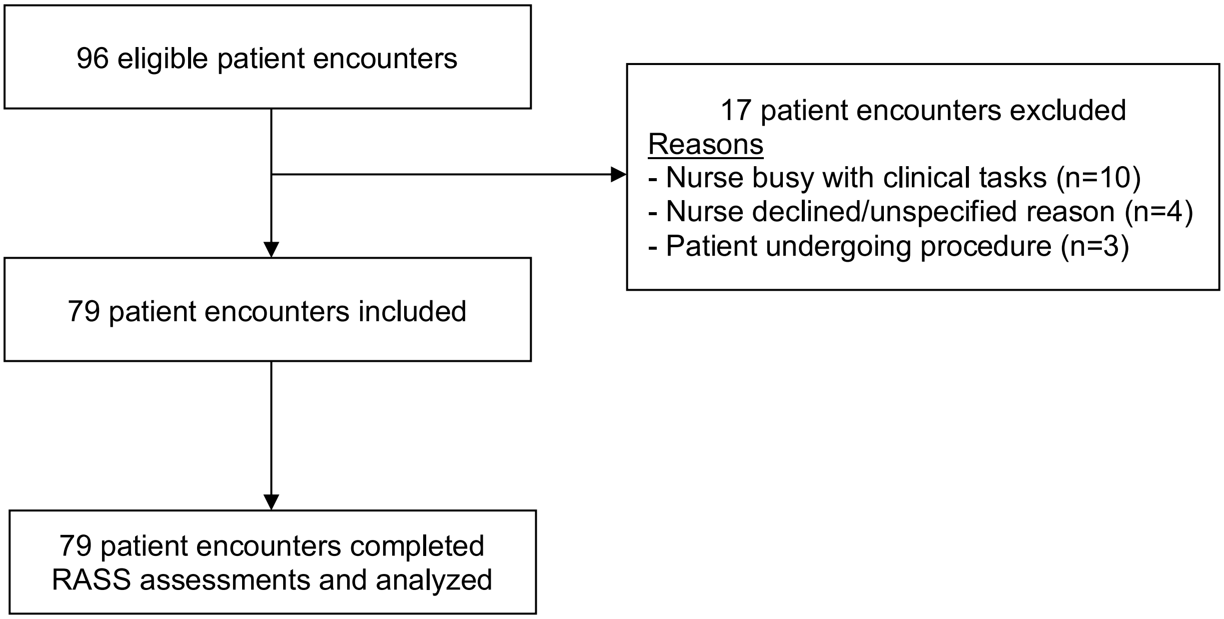

eTable 1. STROBE Checklist

	Item No.	Recommendation	Page No.	Relevant text from manuscript
**Title and abstract**	1	(*a*) Indicate the study's design with a commonly used term in the title or the abstract	1-2	
		(*b*) Provide in the abstract an informative and balanced summary of what was done and what was found	3-4	
**Introduction**				
Background/rationale	2	Explain the scientific background and rationale for the investigation being reported	5	
Objectives	3	State specific objectives, including any prespecified hypotheses	5	
**Methods**				
Study design	4	Present key elements of study design early in the paper	5-6	
Setting	5	Describe the setting, locations, and relevant dates, including periods of recruitment, exposure, follow-up, and data collection	5-7	
Participants	6	(*a*) *Cohort study*—Give the eligibility criteria, and the sources and methods of selection of participants. Describe methods of follow-up *Case-control study*—Give the eligibility criteria, and the sources and methods of case ascertainment and control selection. Give the rationale for the choice of cases and controls *Cross-sectional study*—Give the eligibility criteria, and the sources and methods of selection of participants	5-7	
		(*b*) *Cohort study*—For matched studies, give matching criteria and number of exposed and unexposed *Case-control study*—For matched studies, give matching criteria and the number of controls per case	N/A	
Variables	7	Clearly define all outcomes, exposures, predictors, potential confounders, and effect modifiers. Give diagnostic criteria, if applicable	5-7	
Data sources/ measurement	8*	For each variable of interest, give sources of data and details of methods of assessment (measurement). Describe comparability of assessment methods if there is more than one group	6-7	
Bias	9	Describe any efforts to address potential sources of bias	7	
Study size	10	Explain how the study size was arrived at	8	

Continue on next page

Quantitative variables	11	Explain how quantitative variables were handled in the analyses. If applicable, describe which groupings were chosen and why	8
Statistical methods	12	(*a*) Describe all statistical methods, including those used to control for confounding	8
		(*b*) Describe any methods used to examine subgroups and interactions	N/A
		(*c*) Explain how missing data were addressed	8
		(*d*) *Cohort study*—If applicable, explain how loss to follow-up was addressed<br>*Case-control study*—If applicable, explain how matching of cases and controls was addressed<br>*Cross-sectional study*—If applicable, describe analytical methods taking account of sampling strategy	N/A
		(*e*) Describe any sensitivity analyses	N/A
**Results**			
Participants	13*	(a) Report numbers of individuals at each stage of study—eg numbers potentially eligible, examined for eligibility, confirmed eligible, included in the study, completing follow-up, and analysed	8, eFigure 2
		(b) Give reasons for non-participation at each stage	eFigure 2
		(c) Consider use of a flow diagram	eFigure 2
Descriptive data	14*	(a) Give characteristics of study participants (eg demographic, clinical, social) and information on exposures and potential confounders	8-9, Table 1, eTable 2-3
		(b) Indicate number of participants with missing data for each variable of interest	N/A
		(c) *Cohort study*—Summarise follow-up time (eg, average and total amount)	
Outcome data	15*	*Cohort study*—Report numbers of outcome events or summary measures over time	N/A
		Case-control study—Report numbers in each exposure category, or summary measures of exposure	N/A
		Cross-sectional study—Report numbers of outcome events or summary measures	8, Figures 1-2, Tables 2-3
Main results	16	(*a*) Give unadjusted estimates and, if applicable, confounder-adjusted estimates and their precision (eg, 95% confidence interval). Make clear which confounders were adjusted for and why they were included	8, Figure 1-2, Tables 2-3
		(*b*) Report category boundaries when continuous variables were categorized	N/A
		(*c*) If relevant, consider translating estimates of relative risk into absolute risk for a meaningful time period	N/A

Continue on next page

Other analyses	17	Report other analyses done—eg analyses of subgroups and interactions, and sensitivity analyses	9, Tables 2-3
**Discussion**			
Key results	18	Summarise key results with reference to study objectives	10
Limitations	19	Discuss limitations of the study, taking into account sources of potential bias or imprecision. Discuss both direction and magnitude of any potential bias	13-14
Interpretation	20	Give a cautious overall interpretation of results considering objectives, limitations, multiplicity of analyses, results from similar studies, and other relevant evidence	10-14
Generalisability	21	Discuss the generalisability (external validity) of the study results	13-14
**Other information**			
Funding	22	Give the source of funding and the role of the funders for the present study and, if applicable, for the original study on which the present article is based	1

eTable 2. Richmond Agitation Sedation Scale Assessment Time of Day

Time of day	Medical ICU (50 observations)	Cardiothoracic ICU (29 observations)	Overall (79 observations)
10:00-11:59	11 (22%)	8 (27.6%)	19 (24.1%)
12:00-13:59	14 (28%)	8 (27.6%)	22 (27.8%)
14:00-15:59	12 (24%)	8 (27.6%)	20 (25.3%)
16:00-17:59	9 (18%)	0	9 (11.4%)
18:00-19:59	4 (8%)	5 (17.2%)	9 (11.4%)

ICU = Intensive Care Unit.

eTable 3. Sedative Infusions at the Time of Richmond Agitation Sedation Scale Assessments

	Medical ICU (50 observations)	Cardiothoracic ICU (29 observations)	Overall (79 observations)
Target RASS			
-1 to +1	35 (70.0%)	22 (75.9%)	57 (72.2%)
-3 to -2	6 (12.0%)	4 (13.8%)	10 (12.7%)
-5 to -4	8 (16.0%)	2 (6.9%)	10 (12.7%)
Missing	1 (2.0%)	1 (3.4%)	2 (2.5%)
Number of Sedative Infusions	2 [1-2]	1 [0-2]	1 [0.5-2]
Propofol	25 (50%)	13 (45%)	38 (48%)
Fentanyl	23 (46%)	12 (41%)	35 (44%)
Dexmedetomidine	10 (20%)	5 (17%)	15 (19%)
Midazolam	11 (22%)	0	11 (14%)
Ketamine	6 (12%)	0	6 (8%)
Hydromorphone	4 (8%)	1 (3%)	5 (6%)
Sedative Infusion Rates[1]			
Propofol (mcg/kg/min)	20 [10-40]	20 [15-30]	20 [11.3-40]
Fentanyl (mcg/hr)	50 [25-137.5]	100 [68.8-106.3]	75 [37.5-125]
Dexmedetomidine (mcg/kg/hr)	0.4 [0.3-0.7]	1.5 [1-1.5]	0.7 [0.4-1.1]
Midazolam (mg/hr)	6 [3-10]	N/A	6 [3-10]
Ketamine (mg/kg/hr)	1.9 [1.2-8.1]	N/A	1.9 [1.2-8.1]
Hydromorphone (mg/hr)	2 [1.8-2.5]	0.5 [0.5-0.5]	2 [1-2]
Concurrent NMB[2]	0	0	0

ICU = Intensive Care Unit; N/A = Not available; NMB = Neuromuscular blockade; RASS = Richmond Agitation Sedation Scale.

[1]Sedative infusion rates are summarized as median [IQR] rates among those receiving any (> 0) infusion of corresponding sedatives; those who didn’t receive any of the corresponding sedatives are excluded from the calculation.

[2]One observation in the Medical ICU occurred in a patient with a recent NMB exposure (two hours before the RASS assessment).

eTable 4. Richmond Agitation Sedation Scale Assessments with Comments Provided by Assessors (n=13)

Case No.	MV Reason	Recorded RASS			Comments
		Invest 1	Invest 2	Nurse	
1	Postoperative respiratory failure	-3	-2	-2	"Patient is substantially edematous making eye opening challenging." (Investigator)
2	Acute respiratory failure	-5	-5	-1	"Patient is staring into space with open eyes but not responding to voice or noxious stimulus." (Investigator)
3	Acute respiratory failure	-5	-4	0	"Eyes open at baseline." (Investigator) "Patient is encephalopathic with varying responses to painful stimuli." (Nurse)
4	Acute respiratory failure	-4	-4	-2	"Patient with eyes slightly open but no eye contact, minimally responsive to touch." (Investigator) "Not consistently following commands." (Nurse)
5	Altered mental status	-4	-4	-3	"No eye opening. Patient seemed to be moving even prior to physical stimuli." (Investigator)
6	Altered mental status	-2	-3	-3	"Patient opens eyes to voice intermittently, staring into space, and moving legs restlessly, but appears delirious and does not follow commands." (Investigator) "Patient is agitated and does not follow commands." (Nurse)
7	Acute respiratory failure	-4	-4	-4	"No response to painful stimuli on upper extremities, but responds to painful stimuli to lower extremities." (Investigator)
8	Hemodynamic instability	-4	-4	-4	"Patient did not move to nailbed pinching but responded to tracheal suctioning." (Investigator)
9	Hemodynamic instability	-5	-5	-4	"My RASS assessment was based on response to (tracheal) suction, whereas the investigators did not perform suctioning as part of their assessment." (Nurse)
10	Altered mental status	-3	-3	-2	"Patient swings from +2 to -2." (Nurse)
11	Acute respiratory failure	-3	-3	-2	"Patient inconsistently makes eye contact." (Nurse)
12	Acute respiratory failure	-3	-3	-2	"Patient is able to open eyes and track nurse when stimulated, but becomes restless and agitated when not interacting." (Nurse)
13	Acute respiratory failure	+2	-3	+2	"Patient just received a bolus (sedation) because of agitation." (Investigator)

Invest = Investigator; MV = Mechanical ventilation; RASS = Richmond Agitation Sedation Scale.
ICU type was Cardiothoracic ICU for case numbers 1, 2, and 13; Medical ICU for case numbers 3-12. Target RASS was -1 to +1 for case numbers 1-6, 9-13; -5 to -4 for case numbers 7 and 8.
